# Riparian reserves within oil palm plantations conserve logged forest leaf litter ant communities and maintain associated scavenging rates

**DOI:** 10.1111/1365-2664.12371

**Published:** 2014-12-08

**Authors:** Claudia L. Gray, Owen T. Lewis, Arthur Y. C. Chung, Tom M. Fayle

**Affiliations:** ^1^ Department of Zoology University of Oxford South Parks Road Oxford OX1 3PS UK; ^2^ School of Life Sciences University of Sussex Falmer Brighton BN1 9QG UK; ^3^ Forestry Department Forest Research Centre P.O. Box 1407 90715 Sandakan Sabah Malaysia; ^4^ Faculty of Science, University of South Bohemia and Institute of Entomology Biology Centre of Academy of Sciences Czech Republic Branišovská 31 370 05 České Budějovice Czech Republic; ^5^ Forest Ecology and Conservation Group Imperial College London Silwood Park Campus, Buckhurst Road Ascot Berkshire SL5 7PY UK

**Keywords:** agroecosystems, bait removal, biodiversity conservation, Borneo, Formicidae, riparian buffer, riparian strips, tropical forest fragmentation

## Abstract

1. The expansion of oil palm plantations at the expense of tropical forests is causing declines in many species and altering ecosystem functions. Maintaining forest‐dependent species and processes in these landscapes may therefore limit the negative impacts of this economically important industry. Protecting riparian vegetation may be one such opportunity; forest buffer strips are commonly protected for hydrological reasons, but can also conserve functionally important taxa and the processes they support.
2. We surveyed leaf litter ant communities within oil palm‐dominated landscapes in Sabah, Malaysia, using protein baits. As the scavenging activity of ants influences important ecological characteristics such as nutrient cycling and soil structure, we quantified species‐specific rates of bait removal to examine how this process may change across land uses and establish which changes in community structure underlie observed shifts in activity.
3. Riparian reserves had similar ant species richness, community composition and scavenging rates to nearby continuous logged forest. Reserve width and vegetation structure did not affect ant species richness significantly. However, the number of foraging individuals decreased with increasing reserve width, and scavenging rate increased with vegetation complexity.
4. Oil palm ant communities were characterized by significantly lower species richness than logged forest and riparian reserves and also by altered community composition and reduced scavenging rates.
5. Reduced scavenging activity in oil palm was not explained by a reduction in ant species richness, nor by replacement of forest ant species by those with lower per species scavenging rates. There was also no significant effect of land use on the scavenging activity of the forest species that persisted in oil palm. Rather, changes in scavenging activity were best explained by a reduction in the mean rate of bait removal per individual ant across all species in the community.
6. *Synthesis and applications*. Our results suggest that riparian reserves are comparable to areas of logged forest in terms of ant community composition and ant‐mediated scavenging. Hence, in addition to protecting large continuous areas of primary and logged forest, maintaining riparian reserves is a successful strategy for conserving leaf litter ants and their scavenging activities in tropical agricultural landscapes.

## Abstract 2

Our results suggest that riparian reserves are comparable to areas of logged forest in terms of ant community composition and ant‐mediated scavenging. Hence, in addition to protecting large continuous areas of primary and logged forest, maintaining riparian reserves is a successful strategy for conserving leaf litter ants and their scavenging activities in tropical agricultural landscapes.

## Introduction

Agricultural expansion and intensification continue to cause widespread loss of biodiversity and alter ecosystem functioning. In tropical regions, oil palm is among the most rapidly expanding crops, especially in South‐East Asia (Phalan *et al*. 2013). Palm oil is the world's most widely used vegetable oil; it is used for cooking, cosmetics, detergents and increasingly as a biofuel feedstock. Over the last 10 years, approximately 40% of oil palm expansion in Malaysia and Indonesia (which produce over 80% of global palm oil (FAO 2014)) has been onto forested land (Gunarso *et al*. 2013). As the palm oil industry is now also expanding in Africa and the Neotropics (Butler & Laurance 2010), tropical forests across the globe are likely to be affected by conversion to oil palm plantations.

Conversion of native vegetation to oil palm plantations has profound negative consequences for biodiversity (Savilaakso *et al*. 2014), as well as altering both abiotic and biotic processes. In general, oil palm plantations support <40% of the species found in undisturbed or logged forest (Fitzherbert *et al*. 2008). Oil palm plantations have simplified vegetation structure, as well as higher diurnal temperatures, lower humidity and greater variation in these abiotic variables than forest (Turner & Foster 2006; Luskin & Potts 2011). Carbon sequestration and storage within plantations is lower than in forest (Carlson *et al*. 2012) and rates of soil erosion and frequency of flash flood events are higher (Obidzinski *et al*. 2012).

Nevertheless, oil palm plantations are likely to become an increasingly widespread feature of tropical landscapes, so identifying strategies to reduce the negative impacts of the industry on biodiversity and ecosystem functioning is essential (Foster *et al*. 2011). Limiting the expansion of oil palm onto forested land is very important given the high biodiversity value of this habitat (Edwards *et al*. 2011; Gibson *et al*. 2011; Woodcock *et al*. 2011), but it is also possible that species and processes can be maintained within plantations. In many countries, the vegetation on river banks is protected to help maintain water quality, reduce sedimentation and limit flood risk downstream (Tabacchi *et al*. 2000; Mayer *et al*. 2007). These areas of protected vegetation (also referred to as buffer strips or riparian reserves) can provide non‐crop habitat and movement corridors for species that would not otherwise survive in, or move through, agricultural areas (Marczak *et al*. 2010).

Currently, our understanding of the terrestrial community composition and ecological dynamics of riparian reserves is limited and mainly relates to temperate regions. As a result, existing policy and legislation guiding riparian zone management in the tropics is based on very little ecological information (Ewers *et al*. 2011). To guide policy recommendations and maximize the conservation value of riparian reserves, it is necessary to document their terrestrial biodiversity, how this varies with reserve design, and what impact this has on ecosystem functioning.

Ant communities contribute to many ecological processes, including decomposition, predation and seed dispersal, and form a range of symbiotic relationships with other insects and plants (Lach, Parr & Abbott 2010). Furthermore, since ant communities are reasonably robust to multiple rounds of logging (Woodcock *et al*. 2011), they are a good candidate group for maintaining ecosystem functions in degraded landscapes. The ant fauna in oil palm plantations is less diverse and has a different species composition and spatial structure compared with forested areas (Brühl & Eltz 2010; Fayle *et al*. 2010; Fayle, Turner & Foster 2013; Lucey *et al*. 2014). However, nothing is known about how conversion to oil palm affects the ecological functions carried out by ants. As lower scavenging rates have been found in areas with lower diversity of leaf litter ants, both within tropical forests (Fayle *et al*. 2011) and urban habitats (Tan & Corlett 2012), it is likely that the scavenging activity of ant communities is also altered in oil palm. The scavenging activity of ants influences soil properties; for example, the input of phosphorus from material transported into ant nests can exceed input from decomposing litter in the surrounding soil (Frouz, Santruckova & Kalcik 1997). Hence, changes in the foraging activity of ants are likely to impact on nutrient cycling, soil structure and community composition of other soil arthropods or microbes (Frouz & Jilkova 2008).

We used a novel bait removal method to quantify changes in the scavenging activity of ant communities and individual species across three land uses in Sabah, Malaysian Borneo. We used removal rates of crushed earthworm baits as a surrogate for ant scavenging activity to answer the following questions:

1. How does ground‐foraging ant abundance, species richness and community composition in riparian reserves compare to that in logged forest and oil palm plantations?
2. How does the scavenging activity of ant communities vary across these land uses?
3. Which changes in the ant community explain observed changes in scavenging activity?
4. How does reserve design (width and vegetation structure) affect ant communities and scavenging rates?

## Methods

### Data collection

Study sites were located adjacent to rivers (5–10 m width) within a 600‐km^2^ area of twice‐logged lowland dipterocarp rain forest, acacia plantation *Acacia mangium* and oil palm plantation *Elaeis guineensis* (planted between 1998 and 2012) in Sabah, Malaysian Borneo (117.50 N, 4.60 E). Sampling was conducted in the continuous twice‐logged forest (*n* = 8 sites/streams) in which selective logging had been carried out in the 1970s and 1990s – 2000s (Wearn *et al*. 2013; Luke *et al*. 2014); more details on the logging history of the landscape are given in Struebig *et al*. (2013). The mean basal area of trees at our riparian logged forest sites was 36·1% of that measured by one stream in a nearby primary forest reserve (Maliau Basin). We also sampled in riparian reserves within oil palm (*n* = 9 sites; minimum width of forest on each side of the river = 10 m, maximum = 120 m, mean = 48 m ±26 m SD) and at sites adjacent to rivers in oil palm without a riparian reserve (*n* = 8 sites, Fig. S1). There is no primary forest within 50 km of these sites, and as riparian reserves are isolated linear fragments in a previously logged and then converted landscape, riparian zones in continuous logged forest are an appropriate comparison. All data were collected between April and July, the relatively dry half of the year (Walsh & Newbery 1999), in 2011 (three sampling points per site) and 2012 (12 points per sampling site), to minimize seasonal influences. All sampling points along the riverbank were 30 m apart and within 1 m of the high water line.

At each sampling point, we carried out a 30‐min observation of scavenging activity. In both years, all observations at each site were carried out in 1 day. Baits were pellets made of crushed earthworm (average mass: 0·017 g, maximum diameter: 3 mm; Tropical Fish Food Earthworm Pellets: High Protein, ukfishfood.co.uk). Bait platforms of laminated graph paper were placed with the edge flush with the soil. Each had thirty baits, one placed in the centre of every 2 × 2 cm square (six rows, five columns). We used four different size classes, randomly positioned on the grid for each trial (two × whole pellet, four × half pellet, eight × quarter pellet and 16 × eighth pellet) to ensure that baits were attractive to a range of ant species and similar to naturally occurring sources of protein (e.g. dead invertebrates).

We recorded the time when the first ant entered the bait card and the number of individuals arriving during each five‐minute time period. We recorded temperature at the beginning of each trial as this can affect foraging activity (Ruano, Tinaut & Soler 2000). When a bait item was removed (carried out of its original square), we recorded its position, the time of removal and the species that removed it. Baits were never broken up by ants. Voucher specimens for each species were taken either at the end of the trial or during the trial (if conspecifics were visible and at least 50 cm away from the bait card). Ant voucher specimens were identified to genus, and species where possible using appropriate keys (Bolton 1977; Eguchi 2001; Fisher 2010; Fayle, Yusah & Hashimoto, in prep), the online database AntWeb and reference collections held in the University Museum of Zoology, Cambridge and Natural History Museum, London.

To capture variation in habitat characteristics, we measured humus depth adjacent to the bait card, canopy density (using a spherical densiometer) and tree basal area using the angle point method (Bitterlich 1984). We measured the height of the tallest tree (to the nearest 5 m) within 10 m of the sampling point using a clinometer. We scored understorey vegetation density (for an area of 2 m radius around the bait card and up to 2 m height) and midstorey vegetation density (for an area of 2 m radius around the bait card and 2–4 m height) on an ordinal scale of sparse (fewer than 20 stems or branches) medium (20–60 stems or branches) or dense (few patches of light and 60–100 + stems or branches).

### Analysis

We calculated the following response variables: (i) ant abundance, the total number of foragers arriving at each observation. There were 15 observations in which all bait items were removed before the trial ended, causing subsequent declines in ant abundance. We corrected for this by assuming that ant abundance would have remained at the level observed during the five‐minute period before the last bait item was removed; (ii) ant diversity*,* using the Shannon index; (iii) species count, the number of species arriving at each bait card; and (iv) species richness, the number of species observed across all observations at each site. As we were only able to complete 12 (of 15) observations at two of the 25 sites, we calculated the incidences of each species and then applied a coverage‐based richness estimation technique using the iNEXT online platform (Hsieh, Ma & Chao 2013).

We also calculated functional metrics for each observation: (i) time until the first ant reached the bait card, (ii) number of bait pellets removed, (iii) the proportion of bait mass removed and (iv) recruitment rate. We calculated recruitment rate by extracting the gradient of a linear regression on the number of ants arriving in each 5‐min time period following arrival of the first ant.

We analysed the effect of land use, temperature and their interaction on each of our community and functional metrics. For abundance of ants, diversity of ants, time until the first ant arrived, number of bait pieces removed, proportion of bait mass removed and recruitment rate, we ran generalized linear mixed models (GLMMs). We set site as a random factor and specified transformations and error families as appropriate (see Results). For site‐level species richness, we used a generalized least squares model with land use as a fixed factor and weights to account for heterogeneity of variance (Zuur *et al*. 2009). We tested for differences in community composition using permutational analysis of variance.

There are three non‐mutually exclusive hypotheses for the mechanisms underlying changes in scavenging activity following habitat conversion: (i) that species are lost (and not replaced) when habitats are converted and that this results in reduced bait removal; (ii) that there is turnover in species composition with habitat conversion, and the new species scavenge at a different rate due to a) changes in forager density, b) new species having different scavenging rates to those they replace or c) changes in scavenging rates per forager resulting from increased numerical dominance of species that remove baits relatively slowly; and (iii) that persisting (shared) species alter their scavenging activity in converted habitats (as with hypothesis 2, this could be due to a) changes in the density of foragers, b) changes in the species scavenging activity or c) changes in the numerical dominance of species with different scavenging rates).

To examine whether loss of species explained the observed differences in bait removal (hypothesis 1), we ran a generalized linear model to test for a relationship between the proportion of bait removed (separate analyses for number of bait pieces and proportion of bait mass) and species richness, using data from all sites.

We then tested whether turnover in species could explain differences in bait removal. To ensure that we were only testing for differences between the ant communities responsible for the observed differences in scavenging rates, we combined data from land uses with no significant differences in both ant community composition and overall bait removal rates. We then isolated data for the species that were unique to each group (i.e. the species ‘replaced’ in the change from land use(s) with higher scavenging rates to land use(s) with lower scavenging activity) and tested whether the abundance of foragers differed between these two groups (hypothesis 2 a). To see whether species unique to land uses with less overall scavenging had lower per individual bait removal rates than those they had ‘replaced’ (hypothesis 2 b), we calculated each species’ scavenging rate (the mean bait mass removed per individual visiting the card for each species). We then tested whether the species’ scavenging rates differed between our land‐use groupings. We also calculated the mean bait removal rate per ant (i.e. combining data across all species) and tested whether this differed between species unique to the land uses with high and low rates of scavenging (hypothesis 2 c).

To investigate whether observed differences in scavenging activity could be explained by changes in the persisting species, we first isolated the data on the subset of species found in land uses with both high and low rates of scavenging. We then tested whether the abundance (hypothesis 3 a) and bait removal rate (hypothesis 3 b) of these shared species differed between the land uses. We also tested whether the mean bait removal rate per forager (hypothesis 3 c) differed between land uses.

To investigate effects of riparian reserve width and vegetation structure, we first ran a metric scaling analysis on all vegetation and soil measurements to obtain one numerical index summarizing the greatest variation in these data at each sampling point. The first axis of the output was negatively correlated with understorey density and positively correlated with canopy density, tree height, humus depth, basal area and midstorey density. Since this axis is therefore capturing variation in the 3‐dimensional structure of the vegetation, we refer to it as a vegetation complexity index. We tested for a correlation between vegetation complexity and riparian reserve width and then used GLMMs to test for the effect of width and vegetation complexity on each of the community and function metrics calculated for each observation, specifying year and site as random factors. For the data on species richness calculated at the site level and community composition, we ran a linear model and permanova, respectively, with the mean width for each site, the mean vegetation complexity and their interaction as predictors. All analyses were carried out in R (R Core Team 2014) using the packages vegan (Oksanen *et al*. 2013), lme4 (Bates *et al*. 2014) and nlme (Pinheiro *et al*. 2013).

## Results

### Ant community structure and scavenging rate across land uses

In total, we carried out 366 observations, counted 30 980 individual ants and collected 1906 voucher specimens from which we identified 51 genera and 149 species/morphospecies. A full list of species is given in Table S1.

**Figure 1:**
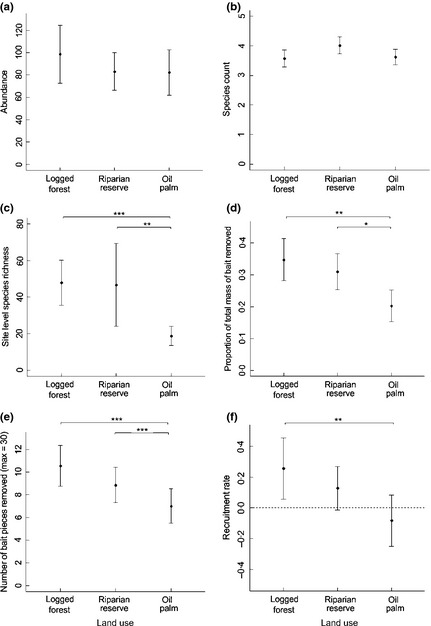
Relationship between land use and (a) abundance of ants arriving at each observation, (b) number of species arriving at each observation, (c) site‐level species richness, (d) the proportion of bait mass removed, (e) number of bait pieces removed and (f) the recruitment rate of ants (dotted line shows no change in number of ants arriving). Plots show mean and 95% CI; stars denote significant differences between groups based on model contrasts (* = *P *<* *0·05, ** = *P *<* *0·01, *** = *P *<* *0·001).

**Figure 2:**
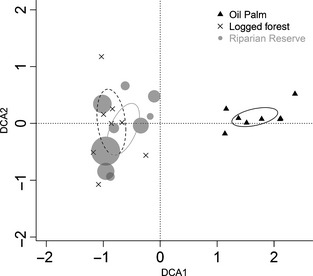
Each point gives the data for incidences of each species across all observations at one site, with the riparian reserve points scaled in proportion to the mean width at each site (min = 23 m, max = 98 m). The ellipses show the standard error of the mean for each land use type. Riparian reserve ant community composition varies with width, but is not significantly different from forest, while both riparian reserve and logged forest differ significantly from oil palm.

There was no significant effect of land use, temperature or their interaction on ant abundance, the number of species at each observation or the Shannon diversity of ants attending the bait (Table 1). Site‐level species richness did not differ between the twice‐logged forest and riparian reserves, but was significantly higher in these land uses than in oil palm (Table 1, Fig. 1a). Community composition also varied across land uses (Table 1, Fig. 2). Multiple comparisons with Bonferroni corrections indicated that riparian reserves had similar community composition to twice‐logged forest, while oil palm differed significantly from the two other land uses.

**Table 1:** Results of GLMMs testing for relationships between land use and ant community or function metrics. Chi‐square, d.f. and *P*‐values are given for likelihood ratio tests of the minimum adequate model against the null model, or the full model against the null model where no fixed factors were significant. *F*, d.f. and *P*‐values are given from linear regression anova tables and permanova (†) tests. Stars denote significance (* = *P* < 0.05, ** = *P* < 0.01, *** = *P* < 0.001)

Model	χ^*2*^	d.f.	*P*
Ant community metrics			
Ant abundance ~ land use + temp + land use: temp	7·1	5	0·216
Species count ~ land use + temp + land use: temp	5·3	5	0·384
Ant diversity (Shannon diversity index) ~ land use + temp + land use: temp	5·0	5	0·419

	*F*	d.f.	*P*
Site‐level species richness ~ land use	15·8	2·22	<0·001***
Community composition ~ land use^†^	19·9	2361	0·001**

	χ^*2*^	d.f.	*P*
Functional metrics			
Time until first ant ~ land use + temp + land use: temp	0·7	5	0·984
Number of bait pieces removed ~ land use	16·7	2	<0·001***
Proportion of bait mass removed ~ land use	10·9	2	0·004**
Recruitment rate ~ land use	6·4	2	0·041*

The time until the first ant arrived did not differ significantly with land use, temperature or their interaction (Table 1). Both bait mass removed and number of baits removed were similar in twice‐logged forest and riparian reserves, and significantly lower in oil palm (Fig. 1d and e). Recruitment rate was significantly higher in twice‐logged forest than in oil palm, with riparian reserve sites intermediate between the two and not significantly different from either (Fig. 1f). Since the twice‐logged forest and riparian reserves did not differ in ant community composition or either measure of scavenging activity, we combined the two forest habitats in our subsequent analyses partitioning the effects of changes in ant communities on scavenging rates.

### Biodiversity–ecosystem function relationships

**Figure 3:**
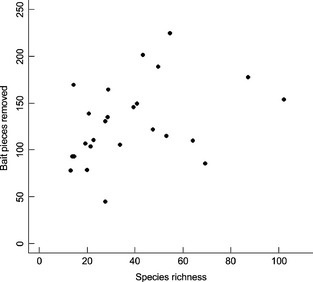
The number of bait pieces removed does not increase significantly with species richness.

There was no significant relationship between the species richness at each site and either number or proportion of bait pieces removed (Table 2, Fig. 3). We found no significant difference between the abundance of foragers or the scavenging activity of the species unique to the forest habitats (twice‐logged forest and riparian reserves) versus the species unique to oil palm that replace them, providing evidence against hypothesis 2 a and 2 b (Table 2). Similarly, we found no significant difference in the abundance of foragers or the scavenging activity of the species shared between forest habitats and oil palm, providing evidence against hypothesis 3 a and 3 b (Table 2). However, the mean bait removal rate per ant was significantly lower for these shared species when they occur in oil palm sites (supporting hypothesis 2 c) and also significantly lower in the species that are unique to the oil palm and replace those unique to the forested land uses (supporting hypothesis 3 c, Table 2).

**Table 2:** Results of GLMMs testing possible mechanisms behind the observed changes in function across land uses. Chi‐square, d.f. and *P*‐values refer to likelihood ratio tests of the model described against the null model. F, d.f. and *P*‐values refer to linear regression anova tables. Stars denote significance ( ** = P < 0.01)

Model	*F*	d.f.	*P*
Hypothesis 1 ‐ changes in scavenging activity due to loss of species			
Bait pieces removed ~ site‐level species richness	3·4	1·23	0·078
Bait mass removed ~ site‐level species richness	2·3	1·23	0·142

	χ^*2*^	d.f.	*P*
Hypothesis 2 – changes in scavenging activity due to turnover in species			
Abundance of foragers ~ higher vs. lower function land uses	0·32	1	0·561
Species’ bait removal rates ~ higher vs. lower function land uses	0·24	1	0·627

	*F*	d.f.	*P*
Bait removal rate per ant ~ higher vs. lower function land uses	11·4	1·23	0·003**

	χ^*2*^	d.f.	*P*
Hypothesis 3 – changes scavenging activity due to changes in species that persist			
Abundance of foragers ~ higher vs. lower function land uses	0·58	1	0·446
Species’ bait removal rates ~ higher vs. lower function land uses	0·07	1	0·798

	*F*	d.f.	*P*
Bait removal rate per ant ~ higher vs. lower function land uses	9·4	1·23	0·005**

### Structural features of riparian reserves

**Figure 4:**
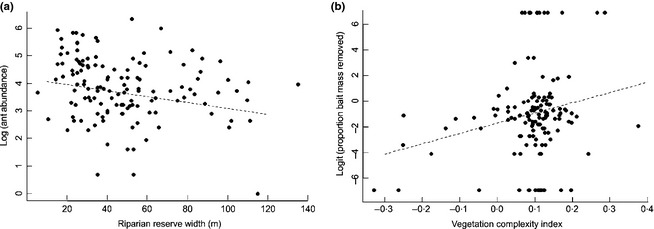
The abundance of individuals (a) visiting the bait card decreases significantly with riparian reserve width and the proportion of bait mass removed (b) increases significantly with vegetation complexity.

There was no significant relationship between riparian strip width and vegetation complexity (χ^2^ = 0·60, d.f. = 1, *P *=* *0·45). Ant abundance declined significantly with increasing riparian strip width, but there were no other significant relationships between reserve width or habitat complexity and the number of species at each observation, the diversity of ants or the site‐level species richness (Table 3, Fig. 4a). Species composition varied significantly with the width of the riparian reserve, but not with the vegetation complexity (Table 3, Fig. 2). There was a significant positive relationship between vegetation complexity and the proportion of bait mass removed, but no other significant relationships between structural features of the riparian reserve and the number of bait pieces taken, the time until the first arrived, or recruitment rate (Table 3, Fig. 4b).

**Table 3:** Results of GLMMs testing for relationships between structural features of riparian reserves and ant community or function metrics. Chi‐square, d.f. and *P*‐values are given for likelihood ratio tests of the minimum adequate model against the null model, or the full model against the null model where no fixed factors were significant. *F*, d.f. and *P*‐values refer to linear regression anova tables and permanova (†) tests. Stars denote significance (* = *P* < 0.05, ** = *P* < 0.01, *** = *P* < 0.001)

Model	χ^*2*^	d.f.	*P*
Abundance ~ width	7·5	1	0·006 ***
Species count ~ width + vegetation complexity	0·6	2	0·735
Ant diversity ~ width + vegetation complexity	3·7	2	0·156
Time until first ant ~ width + vegetation complexity	2·6	2	0·273
Bait pieces removed ~ width + vegetation complexity	4·3	2	0·111
Bait mass removed ~ vegetation complexity	6·3	1	0·012*
Recruitment rate ~ width + vegetation complexity	1·8	2	0·415

	*F*	d.f.	*P*
Site‐level species richness ~ width + vegetation complexity	0·23	2·6	0·805
Community composition ~ width (^†^))	3·1	1129	0·003 **

## Discussion

Oil palm expansion threatens native fauna across the tropics (Fitzherbert *et al*. 2008), but specific management approaches can mitigate the negative impacts of this crop. We found that riparian reserves (strips of native vegetation maintained along rivers) conserve a community of leaf litter ants and level of scavenging activity very similar to that in riparian zones of twice‐logged forest.

### Ant community structure and scavenging rate across land uses

Compared to planting oil palm along the riverbank, the protection of riparian vegetation conserved ant communities similar to those in twice‐logged forest. Riparian areas of oil palm supported only 26% of species found in twice‐logged forest, compared to 64% in riparian reserves. These results are comparable to previous studies from Borneo showing that oil palm plantations contain only 5–19% of the ant species found in primary forest areas (Brühl & Eltz 2010; Fayle *et al*. 2010) and that forest fragments have reduced species richness compared to large areas of forest, but a higher species richness than surrounding oil palm (Brühl, Eltz & Linsenmair 2003; Lucey *et al*. 2014). However, while logged forests retain high conservation value (Edwards *et al*. 2011), neither logged forest nor riparian reserves are as important as primary forest sites for tropical biodiversity conservation (Gibson *et al*. 2011). Many of the species sensitive to disturbance may already have been lost from the twice‐logged forest we use as a reference, so even though we recommend that riparian forest is protected, this should not be prioritized at the expense of continuous forest areas.

Relative to twice‐logged forest, the scavenging activity of leaf litter ant communities was significantly reduced in the oil palm plantations, but maintained in the riparian reserves. This means that animal necromass and other organic material may remain on the soil surface for longer, or be utilized by other consumers, both of which could impact soil nutrient content and soil fauna community structure. As ground‐foraging ants can have a strong influence on the removal of organic matter and the structure of soil in tropical habitats (Frouz & Jilkova 2008; Lach, Parr & Abbott 2010), the impact of this change on soil and leaf litter communities and nutrient cycles may be large and deserves further study.

### Biodiversity–ecosystem function relationships

There was no strong relationship between ant species richness and scavenging activity across the land uses we surveyed, suggesting that other aspects of community change drive shifts in function. Fayle *et al*. (2011) found a slightly stronger relationship between ant species richness and bait removal, but their study was carried out over a smaller spatial scale and did not include agricultural sites. Another factor that could drive changes in function is the abundance of foraging individuals, but we did not find that ant abundance varied significantly with land use.

The presence of particular functionally important species in the forest and riparian reserve sites could also explain higher scavenging rates, but we did not find that oil palm specialists had a lower bait removal rate than the forest specialists they replaced. In addition, the species that persisted in oil palm did not suffer a significant reduction in the amount of bait removed per individual foraging ant, indicating that the reduction in scavenging activity in oil palm is not due to declines in the foraging efficacy of the individual workers from species that persist in the plantations following conversion.

We did find a significant reduction in the mean mass of bait removed per ant in oil palm communities for both the species that persisted and in the oil palm specialists compared to the forest specialists. As our earlier tests show that there is no overall change in the abundance of foraging ants in either of these groups, nor any differences in the species’ bait removal rates, we conclude that the overall reduction in scavenging activity must be due to relative changes in abundance; in oil palm, the species with a lower bait removal rate make up a higher proportion of the ground‐foraging ants compared to forested habitats. We did not collect functional trait data for our species, but changes in the relative abundance of different species may correspond to shifts in functional diversity that affect foraging activity [e.g. body size (Gibb & Parr 2010)], and this would be a promising area for future study.

### Structural features of riparian reserve and management strategies

As riparian reserves can conserve some aspects of biodiversity and ecosystem function within oil palm plantations, it is important to establish management guidelines that will maximize the conservation value of these reserves. We examined two structural features of riparian reserves that could potentially be specified in management protocols: reserve width and vegetation complexity. We found no relationship between species richness and reserve width, which may be because we were sampling riparian fauna that are less sensitive to habitat changes further from the riverbank, or because larger increases in width are required to maintain more sensitive species. However, we did find that the abundance of foraging ants declined with increasing reserve width, which indicates that riparian ant communities are not entirely unresponsive to these changes. It is possible that wider reserves provide a larger foraging area, which reduces ant density without increasing species richness.

The increase in scavenging rate with greater habitat complexity (i.e. greater leaf litter and humus depth) could be because ground cover is necessary for foraging ants to risk carrying baits that reduce their speed and increase their vulnerability to predators. As there was no effect of reserve width on species richness or scavenging rate, we conclude that widths similar to those in this study can conserve leaf litter ant fauna that would be lost if oil palm was planted up to the river bank. We also conclude that riparian reserves can maintain the ant communities, functional role as scavengers (at least within the interior of the riparian vegetation, as our sampling sites were restricted to the centre of the corridor). However, the composition of the wider reserves was more similar to the twice‐logged forest areas (Fig. 2), and so increasing the width of riparian reserves may enhance their conservation value. Importantly, our study only assesses the response of ground‐foraging species, and it is likely that vegetation structure has a much greater impact on arboreal ant species (Widodo *et al*. 2004).

Although we have demonstrated that riparian reserves retain leaf litter ant communities similar to those in larger areas of twice‐logged forest, other aspects of reserve design deserve further attention. We were not able to assess the importance of corridor connectivity, as all the riparian reserves we surveyed were linked to large fragments of logged forest (>3000 ha). Other studies in tropical landscapes have shown that the connectivity to large areas of forest can be crucial for the persistence of some species (Laurance & Laurance 1999; Lees & Peres 2008). It is possible that the riparian sites that we surveyed host sink populations, dependent on immigration of dispersing reproductive individuals from larger areas of forest. However, we only included non‐reproductive individuals in this study, and due to the limited mobility of ground‐foraging ants, we can be confident that all ant species we observed were from established colonies located within the reserves. Nevertheless, satellite image analysis indicates our study landscape was converted from forest within the last 15 years (Hansen *et al*. 2013) and lags in species extinctions as long as 25 years have been documented in tropical forests (Gibson *et al*. 2013). Hence, it is possible that ecological communities within these reserves have not yet reached equilibrium. Longer‐term population studies will be important to determine the extent to which reserves sustain permanent populations.

### Conclusions

Protection of riparian forest can help retain biodiversity and ecological functions within tropical agricultural systems. Our results indicate that the ground‐foraging ant fauna is less diverse in oil palm plantations than nearby forest and that scavenging activity is also impaired; this may have implications for food webs and nutrient cycling in the plantations. Our data suggest that the reduction in scavenging is best explained by increases in the relative abundances of species with low bait removal rates. In contrast, neither the ant communities nor their scavenging activity differed between continuous logged forest and forest in riparian reserves. Hence, we conclude that protecting riparian reserves can help mitigate the negative impacts of oil palm expansion on tropical biodiversity and ecosystem function.

## Supporting information


**Fig. S1.** Map of study sites
**Table S1.** Names of all species observed and the number of foragers counted across all observations in each land use.
