# Supplementary material for: Riparian reserves within oil palm plantations conserve logged forest leaf litter ant communities and maintain associated scavenging rates
Source: J Appl Ecol. 2014 Dec 8;52(1):31–40. doi: 10.1111/1365-2664.12371 (PMC4312969; doi:10.1111/1365-2664.12371)
Supplement: Supplementary file 1 — Fig. S1. Map of study sites Table S1. Names of all species observed and the number of foragers counted across all observations in each land use. [file JPE-52-31-s001.docx]

## Supplementary material

Fig S1 Map of study sites


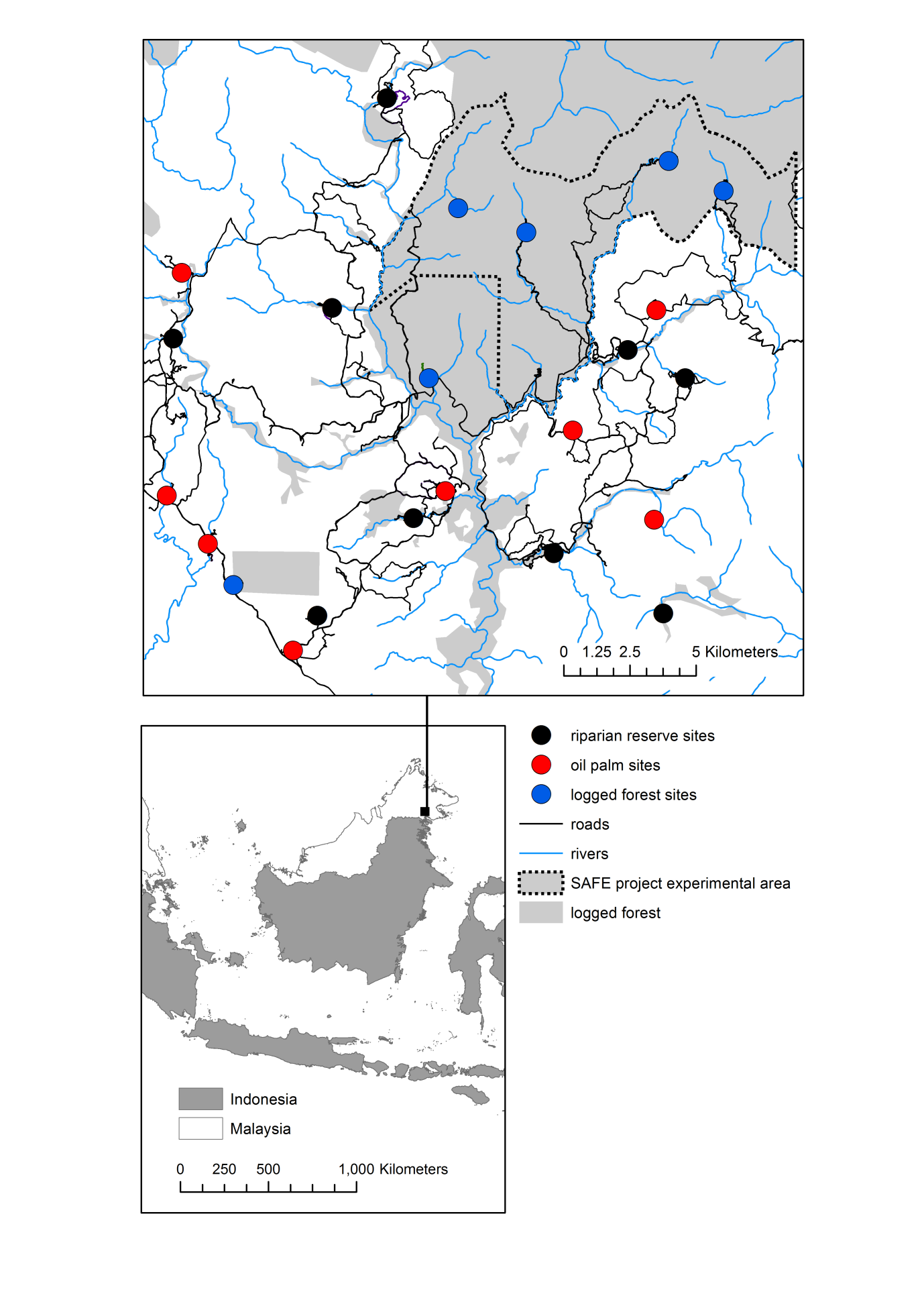


Table S1. Names of all species observed and the number of foragers counted across all observations in each land use. Twenty-two species were found in all land use types, 31 species were found exclusively in logged forest, 27 exclusively in riparian reserves, and 16 exclusively in oil palm. 41 species were found in both riparian reserve and forest sites, eight species were found in only riparian reserve or oil palm sites, and four species were encountered only in forest or oil palm. The non-native species *Anoplolepis gracilipes,* *Tetramorium simillimum* and *Tetramorium bicarinatum* were found only in oil palm, but some individuals of the non-natives *Dolichoderus thoracicus* and *Monomorium ﬂoricola* were also found in the riparian reserves and logged forest.

| **Species name or code** | **Taxonomic Authority** | **Logged Forest** | **Riparian Reserve** | **Oil Palm** |
| --- | --- | --- | --- | --- |
| *Acanthomyrmex* sp.1 |  | 10 | 8 | 0 |
| *Aenictus* sp.1 |  | 1 | 0 | 0 |
| *Anochetus* sp.1 |  | 7 | 0 | 0 |
| *Anoplolepis gracilipes* | Smith 1857 | 0 | 626 | 2347 |
| *Calyptomyrmex* sp.1 |  | 2 | 0 | 0 |
| *Camponotus* sp.1 |  | 1 | 0 | 0 |
| *Camponotus* sp.3 |  | 1 | 1 | 39 |
| *Camponotus* sp.4 |  | 0 | 23 | 0 |
| *Camponotus* sp.5 |  | 0 | 1 | 0 |
| *Camponotus arrogans* | Smith 1858 | 13 | 81 | 0 |
| *Camponotus gigas* | Leach 1825 | 39 | 29 | 0 |
| *Cardiocondyla* sp.6 |  | 0 | 0 | 10 |
| *Cardiocondyla tjibodana* | Karavaiev 1935 | 0 | 0 | 37 |
| *Carebara* sp.1 |  | 0 | 1 | 0 |
| *Carebara* sp.2 |  | 0 | 0 | 1 |
| *Carebara* sp.3 |  | 5 | 0 | 0 |
| *Carebara* sp.4 |  | 0 | 1 | 0 |
| *Crematogaster* sp.5 |  | 3 | 0 | 3 |
| *Crematogaster* sp.6 |  | 0 | 94 | 0 |
| *Crematogaster* sp.7 |  | 2 | 0 | 0 |
| *Crematogaster* sp.9 |  | 0 | 1 | 0 |
| *Crematogaster aff. fraxatrix* | Forel 1911 | 1 | 0 | 0 |
| *Crematogaster baduvi* | Forel 1912 | 6 | 0 | 0 |
| *Crematogaster biroi var.bandarensis* | Forel 1911 | 148 | 202 | 55 |
| *Crematogaster coriaria* | Mayr 1872 | 4 | 107 | 0 |
| *Crematogaster difformis* | Smith 1857 | 1 | 0 | 0 |
| *Crematogaster inflata* | Smith 1857 | 0 | 1 | 0 |
| *Crematogaster longipilosa* | Forel 1907 | 0 | 3 | 154 |
| *Crematogaster modiglianii* | Emery 1900 | 2 | 15 | 0 |
| *Crematogaster rogenhoferi* | Mayr 1879 | 10 | 21 | 62 |
| *Diacamma* sp.3 |  | 1 | 2 | 0 |
| *Diacamma intricatum* | Smith 1857 | 2 | 17 | 1 |
| *Diacamma rugosum* | Le Guillou 1842 | 0 | 8 | 5 |
| *Dolichoderus* sp.2 |  | 2 | 0 | 0 |
| *Dolichoderus thoracicus* | Smith 1860 | 25 | 1 | 0 |
| *Euprenolepis* sp.3 |  | 0 | 6 | 0 |
| *Euprenolepis procera* | Emery 1900 | 6 | 26 | 0 |
| *Euprenolepis thrix* | LaPolla 2009 | 32 | 103 | 0 |
| *Eurhopalothrix* sp.1 |  | 0 | 1 | 0 |
| *Gnamptogenys* sp.1 |  | 2 | 6 | 0 |
| *Gnamptogenys* sp.3 |  | 5 | 1 | 0 |
| *Gnamptogenys aff. binghamii* | Forel 1900 | 6 | 0 | 0 |
| *Hypoponera* sp.1 |  | 1 | 0 | 3 |
| *Iridomyrmex* sp.1 |  | 0 | 0 | 39 |
| *Leptogenys* sp.1 |  | 5 | 5 | 0 |
| *Leptogenys* sp.2 |  | 44 | 42 | 0 |
| *Leptogenys parvula* | Emery 1900 | 0 | 2 | 0 |
| *Leptogenys peuqueti* | André 1887 | 0 | 4 | 3 |
| *Lophomyrmex bedoti* | Emery 1893 | 3378 | 3272 | 1152 |
| *Lophomyrmex longicornis* | Rigato 1994 | 245 | 0 | 0 |
| *Lordomyrma reticulata* | Lucky and Sarnat 2008 | 3 | 0 | 0 |
| *Loweriella* sp.2 |  | 0 | 0 | 29 |
| *Loweriella boltoni* | Shattuck 1992 | 80 | 129 | 59 |
| *Mayriella transfuga* | Baroni Urbani 1977 | 2 | 1 | 0 |
| *Meranoplus* sp.1 |  | 0 | 1 | 0 |
| *Monomorium* sp.2 |  | 0 | 19 | 0 |
| *Monomorium* sp.3 |  | 0 | 3 | 0 |
| *Monomorium* sp.7 |  | 0 | 0 | 1 |
| *Monomorium floricola* | Jerdon 1851 | 2 | 0 | 99 |
| *Monomorium australicum* | Forel 1907 | 0 | 5 | 0 |
| *Myrmecinas* sp.1 |  | 0 | 9 | 1 |
| *Myrmecina* sp.2 |  | 2 | 0 | 0 |
| *Myrmicaria* sp.1 |  | 3 | 0 | 0 |
| *Myrmicaria arachnoides adpressipilosa* | Smith 1857 | 343 | 27 | 0 |
| *Myrmoteras* sp.1 |  | 1 | 0 | 0 |
| *Nylanderia* sp.1 |  | 1 | 15 | 2206 |
| *Nylanderia* sp.2 |  | 22 | 27 | 15 |
| *Nylanderia* sp.3 |  | 147 | 43 | 0 |
| *Nylanderia* sp.4 |  | 25 | 3 | 0 |
| *Nylanderia* sp.5 |  | 0 | 45 | 0 |
| *Nylanderia* sp.6 |  | 86 | 179 | 0 |
| *Nylanderia* sp.7 |  | 26 | 17 | 0 |
| *Odontomachus* sp.1 |  | 4 | 59 | 0 |
| *Odontoponera transversa* | Smith 1857 | 402 | 551 | 127 |
| *Oecophylla* sp.1 |  | 0 | 0 | 3 |
| *Pachycondyla* sp.3 |  | 0 | 3 | 0 |
| *Pachycondyla* sp.4 |  | 0 | 30 | 38 |
| *Pachycondyla* sp.5 |  | 0 | 14 | 0 |
| *Pachycondyla pilidorsalis* | Yamane 2007 | 41 | 33 | 0 |
| *Pachycondyla tridentata* | Smith 1858 | 1 | 0 | 0 |
| *Paraparatrechina* sp.1 |  | 76 | 7 | 12 |
| *Paraparatrechina* sp.2 |  | 131 | 30 | 17 |
| *Paraparatrechina* sp.4 |  | 8 | 15 | 0 |
| *Paraparatrechina minutula var.buxtoni* | Santschi 1928 | 2 | 0 | 0 |
| *Pheidole* sp.1 |  | 4 | 0 | 0 |
| *Pheidole* sp.3 |  | 17 | 140 | 0 |
| *Pheidole* sp.4 |  | 52 | 42 | 0 |
| *Pheidole aff.annexus* | Eguchi 2001 | 80 | 31 | 0 |
| *Pheidole aff.aristotelis* | Forel 1911 | 0 | 52 | 0 |
| *Pheidole aff.deltea* | Eguchi 2001 | 191 | 12 | 0 |
| *Pheidole aff.rabo* | Forel 1913 | 6 | 0 | 0 |
| *Pheidole aff.rugifera* | Eguchi 2001 | 0 | 0 | 63 |
| *Pheidole aglae* | Forel 1913 | 159 | 419 | 47 |
| *Pheidole angulicollis* | Eguchi 2001 | 13 | 23 | 0 |
| *Pheidole annexus* | Eguchi 2001 | 85 | 12 | 0 |
| *Pheidole aristolelis* | Forel 1911 | 9 | 36 | 0 |
| *Pheidole bugi* | Wheeler 1919 | 0 | 0 | 34 |
| *Pheidole butteli* | Forel 1913 | 172 | 105 | 0 |
| *Pheidole cariniceps* | Eguchi 2001 | 275 | 394 | 31 |
| *Pheidole elisae* | Emery 1900 | 3 | 0 | 0 |
| *Pheidole hortensis* | Forel 1913 | 103 | 94 | 0 |
| *Pheidole longipes* | Latreille 1802 | 1 | 30 | 2 |
| *Pheidole lucioccipitalis* | Eguchi 2001 | 371 | 178 | 0 |
| *Pheidole plagiaria* | Smith 1860 | 2 | 200 | 538 |
| *Pheidole quadrensis* | Forel 1900 | 0 | 3 | 0 |
| *Pheidole quadricuspis* | Emery 1900 | 4 | 17 | 0 |
| *Pheidole rabo* | Forel 1913 | 0 | 1 | 0 |
| *Pheidole sarawakana* | Latreille 1802 | 3 | 0 | 0 |
| *Pheidole tawauensis* | Eguchi 2001 | 12 | 3 | 0 |
| *Pheidologeton* sp.1 |  | 2471 | 1028 | 9 |
| *Pheidologeton* sp.4 |  | 1 | 0 | 0 |
| *Pheidologeton pygmaeus* | Emery 1887 | 0 | 1 | 1 |
| *Philidris* sp.1 |  | 17 | 283 | 0 |
| *Plagiolepis* sp.1 |  | 1 | 0 | 13 |
| *Platythyrea* sp.1 |  | 0 | 2 | 0 |
| *Prenolepis* sp.1 |  | 0 | 70 | 0 |
| *Prenolepis* sp.1 |  | 68 | 0 | 0 |
| *Prenolepis* sp.1 |  | 0 | 19 | 0 |
| *Pristomyrmex* sp.1 |  | 0 | 0 | 22 |
| *Proatta* sp.1 |  | 0 | 12 | 1 |
| *Pseudolasius* sp.1 |  | 10 | 1 | 0 |
| *Pyramica* sp.4 |  | 1 | 0 | 0 |
| *Pyramica* sp.5 |  | 1 | 3 | 0 |
| *Recurvidris* sp.2 |  | 175 | 7 | 0 |
| *Recurvidris browni* | Bolton 1992 | 15 | 5 | 0 |
| *Solenopsis geminata* | Fabricius 1804 | 0 | 0 | 601 |
| *Tapinoma* sp.1 |  | 4 | 10 | 724 |
| *Technomyrmex* sp.5 |  | 1 | 0 | 0 |
| *Technomyrmex albipes* | Smith 1861 | 0 | 1 | 0 |
| *Technomyrmex horni* | Forel 1912 | 0 | 1 | 0 |
| *Technomyrmex kraepelini* | Forel 1905 | 64 | 55 | 5 |
| *Tetramorium* sp.1 |  | 27 | 3 | 7 |
| *Tetramorium* sp.11 |  | 5 | 24 | 0 |
| *Tetramorium aff.meshena* | Bolton 1976 | 16 | 26 | 0 |
| *Tetramorium aptum* | Bolton 1977 | 11 | 15 | 2 |
| *Tetramorium bicarinatum* | Nylander 1846 | 0 | 0 | 271 |
| *Tetramorium tortuosum var.eleates* | Forel 1913 | 0 | 0 | 13 |
| *Tetramorium kheperra* | Bolton 1976 | 0 | 0 | 85 |
| *Tetramorium lanuginosum* | Mayr 1870 | 0 | 2 | 0 |
| *Tetramorium longicarinum* | Donisthorpe 1941 | 1 | 0 | 0 |
| *Tetramorium meshena* | Bolton 1976 | 5 | 0 | 0 |
| *Tetramorium pacificum* | Mayr 1870 | 49 | 11 | 1 |
| *Tetramorium parvum* | Bolton 1977 | 0 | 2 | 0 |
| *Tetramorium simillimum* | Smith 1851 | 0 | 0 | 8 |
| *Tetramorium smithi* | Mayr 1879 | 0 | 0 | 179 |
| *Vollenhovia aff.fridae* | Forel 1913 | 1 | 2 | 54 |
